# Whole genome identification and expression analysis of *ARM* gene family in wheat

**DOI:** 10.3389/fpls.2026.1804434

**Published:** 2026-04-24

**Authors:** Jingxu Li, Heng Yue, Xuan Zheng, Yang Yu, Zhenghan Chen, Wanshu Huang, Miaomiao Ma, Quanquan Li, Heng Tang

**Affiliations:** 1 State Key Laboratory of Wheat Improvement, Shandong Agricultural University, Tai’an, China; 2 College of Agronomy, Shandong Agricultural University, Tai’an, China; 3 College of Life Sciences, Shandong Agricultural University, Tai’an, China

**Keywords:** *ARM* gene family, expression pattern, gene duplication events, stress response, synteny analysis, wheat

## Abstract

Wheat is one of the world’s major food crops, and its yield and quality are significantly affected by various biotic and abiotic stresses. Armadillo (ARM) repeat proteins regulate plant development, signaling, and stress responses; however, the *ARM* gene family in wheat remains uncharacterized. In this study, we identified ARM genes from six plant species: *Triticum aestivum, Triticum urartu, Aegilops tauschii, Triticum dicoccoides, Arabidopsis thaliana*, and *Oryza sativa* ssp. *japonica*, totaling 32, 8, 13, 22, 15, and 8 genes, respectively. These genes were classified into three subfamilies (C1-C3). We analyzed their gene structures, conserved motifs, predicted protein properties, and phylogeny. Genomic analyses were conducted to investigate gene family expansion and evolutionary selection. Cis-acting element analysis and RNA-seq were used to assess stress responsiveness, which was further validated by qRT-PCR. Genomic analyses indicated that polyploidy-driven segmental duplications expanded the wheat *ARM* gene family, with purifying selection dominating its evolution. Cis-acting element analysis revealed the molecular basis for *TaARM* gene responses to abiotic stress. RNA-seq and qRT-PCR analyses demonstrated distinct response characteristics among *TaARM* members. Specifically, *TaARM13* and *TaARM22* responded to biotic stresses (powdery mildew and stripe rust); *TaARM18* was induced by powdery mildew and low-temperature stress; and *TaARM26* was specifically upregulated by low-temperature stress. These results suggest that different members of the *TaARM* gene family play unique and specific roles in coping with various stresses. Future research integrating multi-omics data and molecular validation will support the breeding of stress-resilient wheat varieties.

## 1 Introduction

Repetitive protein domains perform essential functions in plants. There are some repetitive protein domains including armadillo (ARM) (Gumber et al., 2019), tetratricopeptide (TPR) (Zhang et al., 2025a), leucine-rich (LRR) (Isaksson et al., 2025), and ankyrin (ANK) repeats (Raghuraman and Park, 2024) have been reported before. These domains facilitate protein-protein interactions and are involved in a range of fundamental plant processes, such as growth and development, signal transduction, cytoskeleton organization, and nuclear transport. They have also made significant contributions to the stress responses of plants (Sharma and Pandey, 2016). Among these, ARM repeats were originally identified in the Drosophila segment polarity gene product armadillo (Riggleman et al., 1989). Structurally, each ARM repeat unit comprises approximately 42 amino acids that fold into three α-helices (H1-H3) (Huber et al., 1997). To date, 56, 54, 169, 158, and 108 *ARM* members have been characterized in maize (*Zea mays*), potato (*Solanum tuberosum*), upland cotton (*Gossypium hirsutum*), rice (*Oryza sativa*), and *Arabidopsis thaliana*, respectively, with expression analyses completed (Mudgil et al., 2004; Sharma et al., 2014; Yu et al., 2023; Liu et al., 2024a, 2024).

ARM repeat proteins perform diverse functions in plants, primarily reflected in plant-specific developmental processes, growth regulation-related signal transduction, and responses to biotic and abiotic stresses (Samuel et al., 2006). For example, the ARM repeat gene *ZAK IXIK* in *Arabidopsis thaliana* promotes early embryo and endosperm development through a unique gametophytic maternal effect, thereby influencing post-fertilization embryo and endosperm growth (Ngo et al., 2012). The *VPNB1* gene in *Arabidopsis* encodes an ARM repeat protein involved in vascular tissue development (Podia et al., 2018). In maize, the *ZmARM4* locus affects grain filling and seed weight and is closely associated with crop yield (Kong et al., 2019). Regarding stress responses, *GhARM144* in upland cotton regulates resistance to *Verticillium dahliae* via interaction with *GhOSM34* (Liu et al., 2024a). ARM-repeat proteins in tomato contribute to resistance against *Tomato leaf curl New Delhi virus* (ToLCNDV) (Mandal et al., 2017). The potato *StPUB17* gene represents a novel UND/PUB/ARM repeat-type gene associated with late blight resistance and NaCl stress (Ni et al., 2010). In addition, plant U-box Armadillo repeat (PUB-ARM) ubiquitin ligases play important roles in defense by mediating ubiquitination of target proteins (Knop et al., 2021).

Wheat, one of the world’s major staple crops, plays a vital role in global food production (Shewry and Hey, 2015). However, its yield and quality are constrained by various biotic and abiotic stresses, including powdery mildew, stripe rust, low-temperature stress, and salinity stress (Ashraf, 2014; Ma et al., 2021a; Xiao et al., 2022). While studies have shown that partial duplication of the *HvARM1* gene in barley enhances resistance to powdery mildew through a neofunctionalization mechanism (Rajaraman et al., 2018), and the stress-responsive functions of the *ARM* gene family have been systematically elucidated in plants such as *Arabidopsis thaliana* and maize, a systematic investigation and characterization of the *ARM* gene family in gramineous crops, particularly in wheat, remain largely unexplored.

In this study, members of the *ARM* gene family in wheat and its progenitor species were identified at the genome-wide level, followed by detailed bioinformatic analyses of their chromosomal locations, predicted subcellular localization, physicochemical properties, gene structures, conserved motifs, phylogenetic relationships, cis-acting regulatory elements, gene duplication events, synteny relationships, and expression levels across different tissues. Using publicly available RNA-seq data, the expression levels of wheat *ARM* gene family members under specific biotic/abiotic stresses were analyzed, and the expression responses of selected *TaARM* genes to these stresses were further experimentally validated. This study lays a solid foundation for further exploration and functional characterization of stress-responsive *TaARM* genes in wheat and their application in breeding for stress resistance.

## 2 Materials and methods

### 2.1 Plant materials and treatments

Fielder was used as the experimental material in this study. Plants were grown in a controlled environment growth chamber under a 16 h light/8 h dark photoperiod at 24/20 °C with approximately 70% relative humidity. Treatments began at the one-leaf-one-heart stage, using leaves of uniform vigor. For low-temperature stress, young leaves were harvested at 0, 24, 48, and 72 h after transfer to 4 °C. For stripe rust, leaves were inoculated with CYR32 and sampled at the same intervals. For powdery mildew, samples were taken at 0, 24, 48, and 72 h post-treatment. For salt stress, roots were collected at 0 and 7 days after treatment with 200 mmol/L NaCl. Three biological replicates were collected per time point. All samples were frozen in liquid nitrogen and stored at -80°C.

### 2.2 Identification of ARM family members

Genome sequences, GFF3 annotations, and complete protein sets for *Triticum aestivum* (Chinese Spring, IWGSC_RefSeq v1.0), *Triticum urartu* G1812 (AA, G1812 Tu_2.0), *Aegilops tauschii* AL8/78 (DD, Aet_v4.0), *Triticum dicoccoides* (Wild emmer wheat Zavitan, AABB, WEWSeq_v1.0), *Arabidopsis thaliana* (TAIR 10), and *Oryza sativa* ssp. *japonica* (rice, IRGSP-1.0) were obtained from the Ensembl plants database (https://plants.ensembl.org/index.html). The Hidden Markov Model (HMM) profile of the ARM domain (PF00514) was downloaded from the InterPro database (https://www.ebi.ac.uk/interpro/).

ARM family members were identified by searching the whole-genome protein sequences of common wheat and its progenitors using the HMM profile via the Simple HMM Search tool in TBtools software (https://github.com/CJ-Chen/TBtools; E−value ≤ 1e−5). Putative ARM family members were validated against the InterPro database. InterPro uses predictive models, known as signatures, provided by several different databases (referred to as member databases) that make up the InterPro consortium (Blum et al., 2025). Only those containing the ARM domain (SMART: arm_5; Pfam: Armadillo/beta−catenin−like repeat; E−value ≤ 1e−3) were retained. The same pipeline was applied to A. *thaliana* and rice. **Supplementary Table 6** provides a detailed record of the output results from the Simple HMM Search and InterPro database (including SMART and Pfam).

### 2.3 Phylogenetic analysis of *ARM* gene family

A combined phylogenetic tree was constructed from the full-length amino acid sequences of 98 ARM family members across six species. Sequence alignment and phylogenetic analysis were performed using the ClustalW algorithm in MEGA 12.0 software (https://www.megasoftware.net/home) with default parameters. The phylogenetic tree was constructed using the Maximum Likelihood method with 1000 bootstrap replicates, the Poisson model, and partial deletion for gap handling (Liu et al., 2024b). The resulting tree was interactively visualized and refined using the online tool iTOL (https://itol.embl.de/).

### 2.4 Gene structure and conserved motif analysis of the *ARM* family members across six species

Gene structure information for 98 *ARM* gene family members was extracted from GFF3 annotation files of six species. Their full-length amino acid sequences were then analyzed for conserved motifs using the online MEME Suite 5.5.8 (https://meme-suite.org/meme/) with the following parameters: Maximum number of motifs=10; Motif width=6–50; other settings were kept as default. Gene structures and conserved motifs of all 98 members were subsequently visualized using the Gene Structure View (Advanced) tool in TBtools.

### 2.5 Prediction of cis-acting regulatory elements in *TaARM* genes

Using TBtools software, the sequences of 2000 bp upstream of the transcription start site (TSS) and annotation information of the *TaARM* gene were extracted from the wheat genome file and the GFF3 annotation file, and the results were submitted to the PlantCare database (https://bioinformatics.psb.ugent.be/webtools/plantcare/html/) (Lescot et al., 2002) to predict cis-acting regulatory elements. Visualization was carried out using TBtools and Excel. Specifically, TBtools was used to generate heatmaps and sequence distribution maps of cis-acting regulatory elements, and Excel was used to produce stacked bar charts.

### 2.6 Physicochemical properties of ARM family members across six species

For the full-length sequences of 98 ARM family members, amino acid length (aa), theoretical isoelectric point (pI), molecular weight (kDa), and instability index were characterized using the Protein Parameter Calculator implemented in TBtools. Furthermore, subcellular localization of these proteins was predicted with the online tool WoLF PSORT (https://wolfpsort.hgc.jp/).

### 2.7 Chromosome distribution and gene duplication events

Chromosomal positions of the *ARM* genes were annotated using GFF3 files from *T. aestivum* and its progenitors (*T. dicoccoides*, *T. urartu*, and *Ae. tauschii*). To visualize their genomic distribution, physical location maps were generated via the Gene Location Visualize tool in TBtools, which displays the arrangement and relative spacing of genes on each chromosome.

Identification of *TaARM* gene duplication events and wheat-related syntenic regions was performed using the One Step MCScanX tool in TBtools (the process uses the tool’s default parameters). To quantify selection pressures, the *Ka* (non-synonymous substitution rate), *Ks* (synonymous substitution rate), and *Ka/Ks* ratios for syntenic gene pairs were calculated using the Simple Ka/Ks Calculator (NG) in TBtools. The genomic distributions of duplication events and synteny were visualized with TBtools’ Advanced Circos and Multiple Synteny Plot functions, respectively. Subsequent statistical analysis and custom plotting of *Ka/Ks* ratios were carried out using R. *Ka/Ks* ratios were interpreted as follows: < 1 (purifying selection), = 1 (neutral selection), and > 1 (positive selection) (Vitti et al., 2013).

### 2.8 Expression pattern analysis of *TaARM* gene family

RNA-seq data for Chinese Spring were obtained from the WheatOmics database (http://wheatomics.sdau.edu.cn/expression/index.html) (Ma et al., 2021b). This included expression profiles from different tissues during growth and development (IWGSC, 2014), as well as under various biotic (powdery mildew and stripe rust; Zhang et al., 2014) and abiotic (cold and salt; Li et al., 2015; Zhang et al., 2016) stress conditions. To analyze the expression patterns of the *TaARM* family members, a heatmap was generated using the Heatmap tool in TBtools software. Row cluster analysis was performed based on the log_2_-transformed transcripts per million (TPM) values of the *TaARM* genes. log_2_(TPM + 1) > 3 indicates high gene expression levels, whereas 0 ≤ log_2_(TPM + 1) < 1 indicates low or no gene expression.

### 2.9 qRT-PCR analysis

To validate the expression levels of *ARM* genes, six genes were selected for quantitative real-time PCR (qRT−PCR) analysis. Gene−specific primers were designed using Primer Premier 5 software, and their specificity was verified against the reference genomes of Chinese Spring and Fielder (Sato et al., 2021; Zhu et al., 2021). Total RNA was isolated using the Trizol method and quantified spectrophotometrically. First-strand cDNA was synthesized with the Hiscript IV ALL-in-One Ultra RT SuperMix for qPCR (Vazyme). qRT-PCR assays were conducted on a StepOne detection system using the 2× ChamQ Blue Universal SYBR qPCR Master Mix (Vazyme). Each 20 μL reaction contained 10 μL of 2× Master Mix, 2 μL of each primer, 1 μL of cDNA template, and 5 μL of RNAse-free water. The thermal cycling protocol included an initial denaturation at 94 °C for 3 min, followed by 40 cycles of denaturation at 94 °C for 10 s and annealing/extension at 60 °C for 30 s with fluorescence acquisition. All reactions were run in triplicate, with the gene *TaEF-1α* serving as an internal reference. All primers were synthesized by Sangon Biotech (Shanghai) Co., Ltd. Relative gene expression levels were calculated using the $2^{-\Delta \Delta \text{Ct}}$ method. Expression data for the *TaARM* genes were visualized with GraphPad Prism 9.0.2 software. Statistical significance between control and treatment groups was assessed using the independent Student’s t-test. Statistically significant differences between control and treatment groups are denoted by asterisks: ns (*p* ≥ 0.05), * (*p* < 0.05); ** (*p* < 0.01). The primer sequences used for qRT-PCR are provided in **Supplementary Table 4**.

## 3 Results

### 3.1 Identification and phylogenetic analysis of *TaARM* genes

The complete protein sequences of *ARM* gene family members from *T. aestivum*, *T. urartu*, *Ae. tauschii*, *T. dicoccoides*, *A. thaliana*, and *O. sativa* ssp. *japonica* were retrieved and screened using TBtools software. Conserved domain analysis identified 32, 8, 13, 22, 15, and 8 ARM members in *T. aestivum*, *T. urartu*, *Ae. tauschii*, *T. dicoccoides*, *A. thaliana*, and *O. sativa* ssp. *japonica*, respectively. Unlike previous studies that frequently adopted relatively loose thresholds (E−value ≤ 1), this research adopted a unified approach, employed relatively stringent threshold settings which implemented a rigorous initial HMM search cutoff (E−value ≤ 10^-5^) and a subsequent validation threshold against the canonical InterPro ARM domain (PF00514) with an E−value ≤ 10^-^³ and defined the categories of *ARMs* during the screening of *ARM* gene family members across six species. Consequently, the number of identified *ARM* gene family members in *A. thaliana* and rice exhibited significant difference compared with the results reported in prior work (Mudgil et al., 2004; Sharma et al., 2014). Accordingly, the number of reported ARM family members was substantially reduced: from 108 to 15 in *Arabidopsis thaliana* and from 158 to 8 in rice (**Supplementary Table 9**). Physicochemical properties of the TaARM proteins were analyzed with the Protein Parameter Calc tool in TBtools. The protein lengths ranged from 176 to 957 amino acids (aa), molecular weights from 19.50 to 101.10 kDa, theoretical isoelectric points (pI) from 4.73 to 8.36, and instability indices from 29.14 to 64.90. Among these, 81.25% of the proteins were acidic (pI < 7), 12.50% were neutral (pI ≈ 7), and 6.25% were alkaline (pI > 7). Subcellular localization predictions indicated that the TaARM proteins were distributed in the chloroplast (65.6%), cytoplasm (12.5%), nucleus (18.8%), endoplasmic reticulum (9.4%), and plasma membrane (6.3%). Some TaARM proteins were predicted to localize to multiple subcellular compartments, resulting in the cumulative sum of these predictions exceeding 100%. For instance, TaARM10 is predicted to be dually localized in the chloroplast and nucleus. Detailed physicochemical properties and subcellular localization information for these species are provided in **Supplementary Table 1**.

Based on their chromosomal order and physical position, the *ARM* members were systematically named from 1 to the total count per species: *TaARM1–TaARM32* for common wheat (*T. aestivum*), *TuARM1–TuARM8* for *T. urartu*, *AetARM1–AetARM13* for *Ae. tauschii*, *TdARM1–TdARM22* for *T. dicoccoides*, *AtARM1–AtARM15* for *A. thaliana*, and *OsARM1–OsARM8* for *O. sativa* ssp. *japonica*.

**Figure 1:**
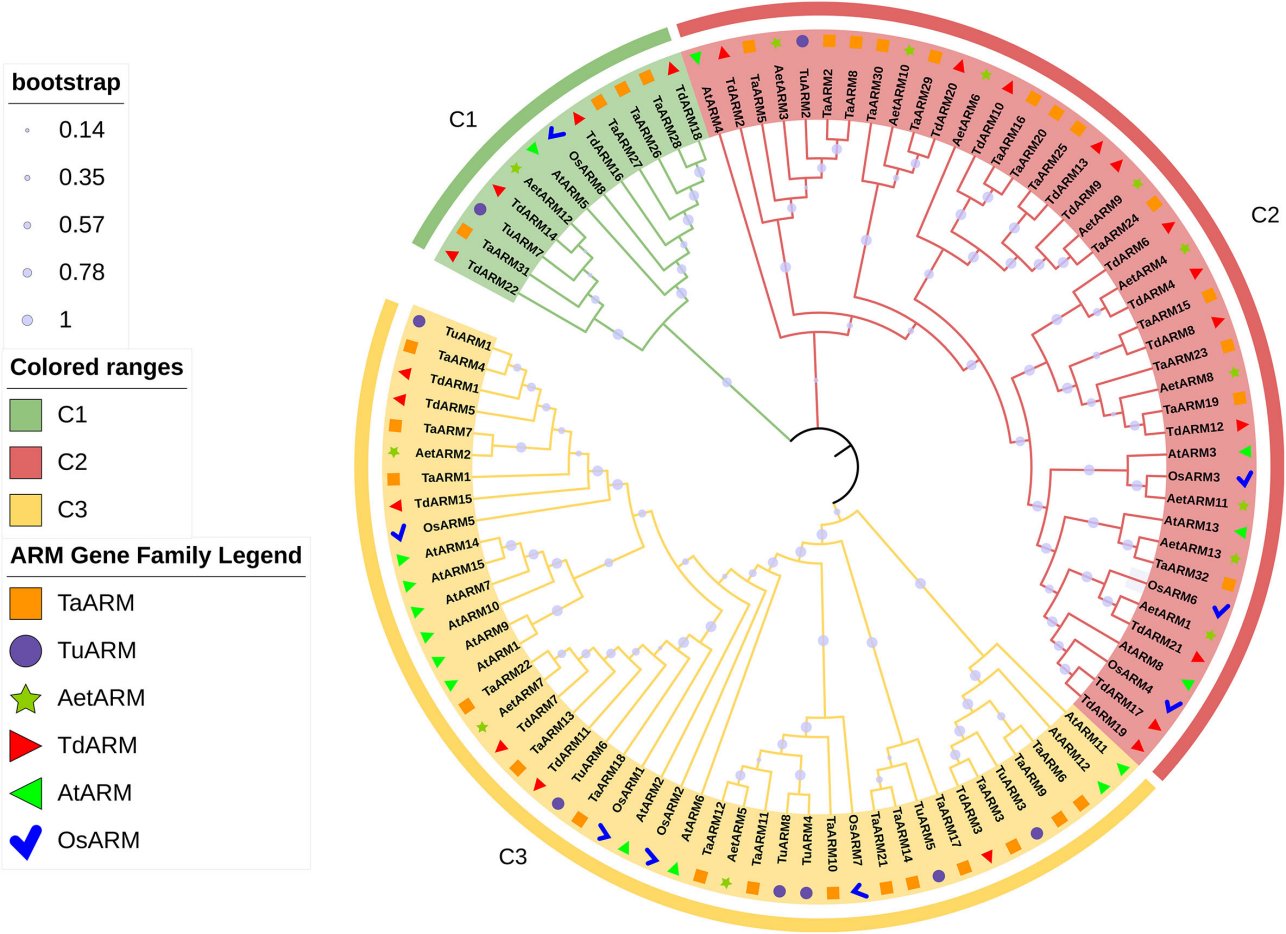
Phylogenetic analysis of *ARM* genes in *T. aestivum, T. urartu, Ae. tauschii, T. dicoccoides, A. thaliana*, and *O. sativa* ssp. *japonica*. The phylogenetic tree depicts the relationships among 32 *TaARM* (orange square), 8 *TuARM* (purple circle), 13 *AetARM* (green pentagram), 22 *TdARM* (red triangle), 15 *AtARM* (green triangle), and 8 *OsARM* (blue check mark) genes. Different groups are distinguished by distinct branch colors, with a consistent background color within each group to denote different *ARM* gene types (C1, green; C2, red; C3, yellow). The bootstrap value of each branch is represented by the size of the circle.

**Figure 2:**
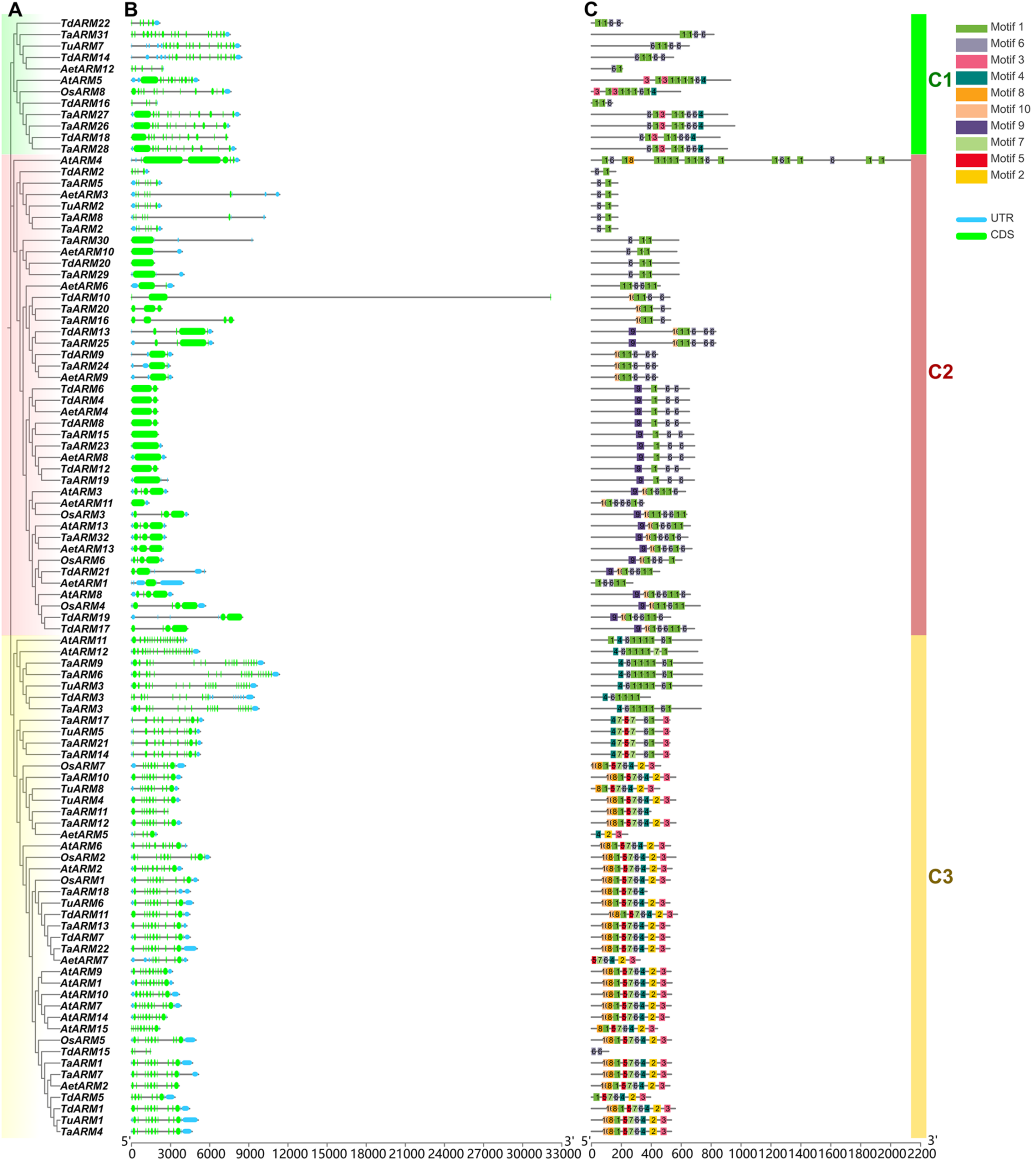
Phylogenetic relationships, gene structure, and conserved motifs of the *ARM* genes. **(A)** The phylogenetic tree, identical to the one in **Figure 1**, is displayed using the Cladogram tree layout. **(B)** Exon/intron structure of the *ARM* genes. **(C)** Distribution of conserved motifs in the *ARM* genes.

To investigate the phylogenetic evolution of TaARM proteins, a total of 98 ARM sequences—32 TaARM, 8 TuARM, 13 AetARM, 22 TdARM, 15 AtARM, and 8 OsARM—were subjected to phylogenetic analysis using MEGA 12.0. The resulting tree revealed that all members clustered into three distinct subfamilies (C1, C2, and C3). Among the TaARM proteins, four (TaARM26, TaARM27, TaARM28, and TaARM31) grouped within the C1 subfamily; thirteen (TaARM2, TaARM5, TaARM8, TaARM15, TaARM16, TaARM19, TaARM20, TaARM23, TaARM24, TaARM25, TaARM29, TaARM30, and TaARM32) belonged to C2; and the remaining fifteen (TaARM1, TaARM3, TaARM4, TaARM6, TaARM7, TaARM9, TaARM10, TaARM11, TaARM12, TaARM13, TaARM14, TaARM17, TaARM18, TaARM21, and TaARM22) fell into C3 (**Figures 1**, **2A**). For the other five species, the distribution of proteins across the three subfamilies was as follows: TuARM showed a ratio of C1:C2:C3 = 1:1:6; AetARM, 1:9:3; TdARM, 4:12:6; AtARM, 1:4:10; and OsARM, 1:3:4.

### 3.2 Chromosomal localization of *ARM* gene family members

**Figure 3:**
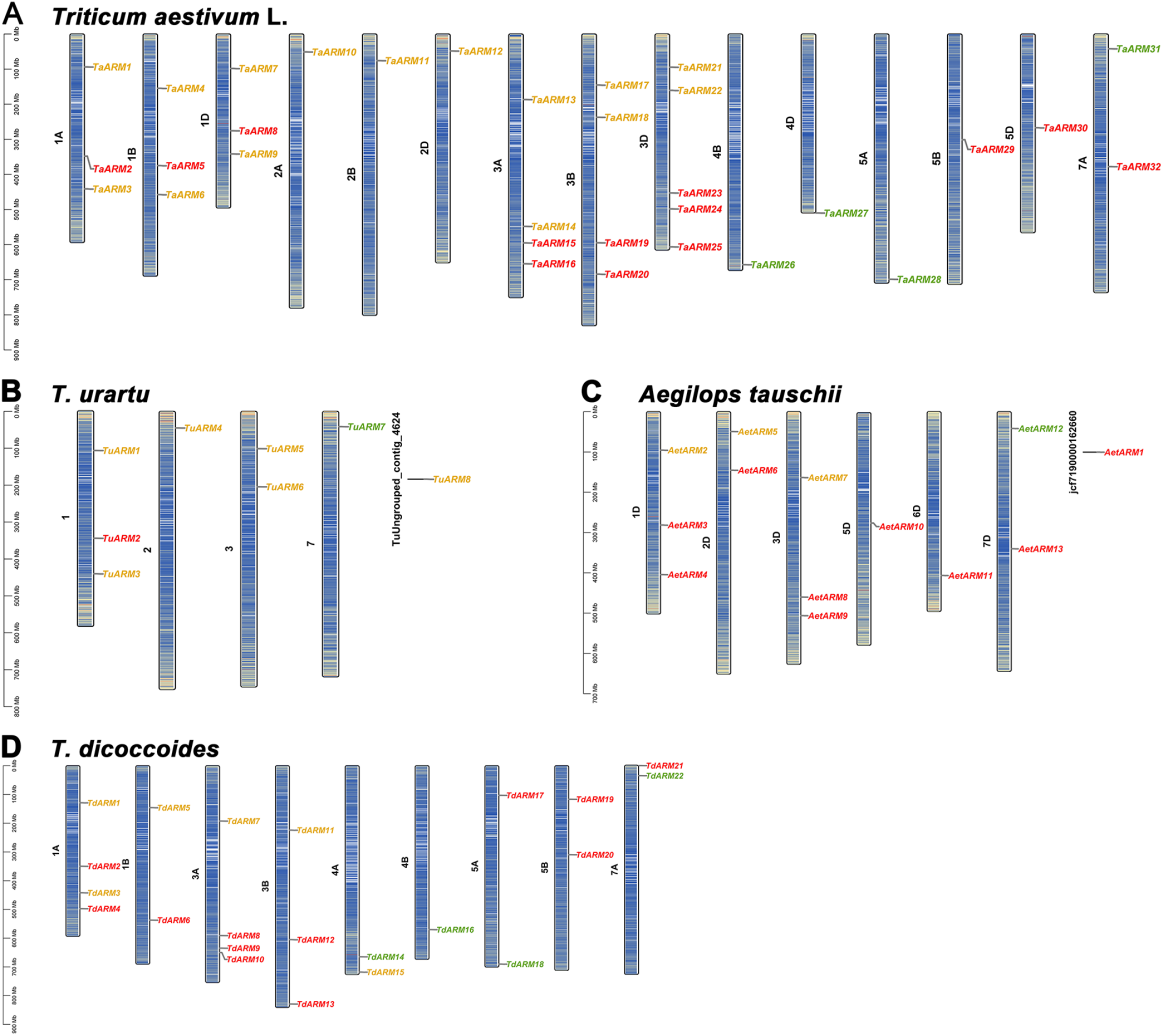
Chromosomal distribution of *ARM* genes in wheat and its progenitors. *ARM* genes of the C1, C2, and C3 subfamilies are marked with green, red, and yellow, respectively. Chromosomal regions are color-coded such that deeper color indicates higher gene density. **(A–D)** show the chromosomal distributions of *ARM* genes in *T. aestivum, T. urartu, Ae. tauschii*, and *T. dicoccoides*, respectively. **(B)** The chromosomal locations of TuUngrouped_contig_4624 (*TuARM8*) and **(C)** jcf7190000162660 (*AetARM1*) remain undetermined.

Based on the GFF3 annotation files of *T. aestivum*, its progenitors (*T*. *urartu*, *Ae. tauschii*, *T. dicoccoides*), *A. thaliana*, and *O. sativa* ssp. *japonica*, the chromosomal locations of *ARM* gene family members were determined. The 32 *TaARM* genes were unevenly distributed across 15 wheat chromosomes, with the highest abundance (5 genes) on chromosome 3D and the lowest (1 gene each) on chromosomes 2A/2B/2D, 4B/4D, and 5A/5B/5D (**Figure 3A**). The distribution ratio of *ARM* genes among the A, B, and D subgenomes of wheat was 11:10:11.

In *T. urartu*, the 7 *TuARM* genes were unevenly located on chromosomes 1, 2, 3, and 7 (with *TuARM8* not assigned to a chromosome). In *Ae. tauschii*, the 12 *AetARM* genes were unevenly distributed across chromosomes 1D, 2D, 3D, 5D, 6D, and 7D (*AetARM1* was not assigned to a chromosome). In *T. dicoccoides*, the 22 *TdARM* genes were unevenly scattered over 9 chromosomes. Across these four species, *ARM* family members on the same chromosome were generally separated by considerable physical distances, suggesting that tandem duplication was unlikely to drive the expansion of this gene family.

In wheat, C1 subfamily members were predominantly localized approximately 100 Mb from telomeric regions. C2 subfamily members were distributed across middle or near−mid chromosomal regions as well as near telomeres. According to **Supplementary Table 1**, the chromosomal localization of C3 family genes in wheat and its ancestral relatives is strongly biased toward chromosomes 1 to 3 (1A, 1B, 1D, 2A, 2B, 2D, 3A, 3B, and 3D). So, the distribution pattern of the C3 subfamily was largely conserved across the wheat progenitors: *T. urartu* (AA), *Ae. tauschii* (DD), and *T. dicoccoides* (AABB), with the exception of *TdARM15*, which was localized to chromosome 4A in *T. dicoccoides* (**Figures 3B–D**).

### 3.3 Analysis of gene structure and conserved motifs of *TaARM* family members

The divergence in exon-intron organization among gene families often reflects their distinct evolutionary trajectories, and comparative analysis of such structures is crucial for elucidating evolutionary and functional relationships among genes within plant species (Wang et al., 2013). Based on the wheat GFF3 annotation file, genomic annotations for the 32 family members were extracted, determined the exon-intron structure of each *ARM* gene, and constructed corresponding gene models using TBtools (**Figure 2B**). Substantial variation in exon number was observed among *TaARM* genes, ranging from 1 to 19. *TaARM3*, *TaARM6*, and *TaARM9* contained the highest number of exons (19 each), together representing 9.38% of the family. In contrast, *TaARM15* and *TaARM23* possessed only one exon, accounting for 6.25% of all members. The largest group consisted of nine genes (28.13%), each containing 11 exons.

Using MEME Suite 5.5.8, a total of 10 conserved motifs were identified from the analysis of 98 ARM proteins derived from wheat, its progenitor species, *A. thaliana*, and rice (**Figure 2C**; **Table 1**). Among these motifs, the shortest comprised 29 amino acids, while the longest consisted of 50 amino acids. The sequence logos of the conserved motifs identified in the TaARMs proteins are shown in **Supplementary Table 2**.

**Table 1:** List of the putative motifs identified in ARM proteins.

Motif	Width	Sites	Best possible match
Motif 1	41	98	SENRKVVAECGAVPJLVKLLSSPDADVREQAVWALGNLAID
Motif 2	50	28	FDIKKEAAWAISNATSGGSHDQIKYLVSQGCIKPLCDLLICPDPRIVTVC
Motif 3	41	40	YAQMIDEAEGLEKIENLQSHDNNEIYEKAVKILESYWLEEE
Motif 4	35	46	DHGALPCLLNLLTRNHKKSIKKEACWTISNITAGN
Motif 5	29	35	LAQLNEHAKLSMLRNATWTLSNFCRGKPQ
Motif 6	29	98	GVIPPLVELLLHGSPSVLKPALRALGNJA
Motif 7	41	35	EQVKPALPALARLIHSADEEVLTDACWALSYLSDGTNDKIQ
Motif 8	41	29	RSPPIEEVIQSGVVPRFVEFLTREDYPQLQFEAAWALTNIA
Motif 9	50	22	SGQTYERESIQRWFDSGKSTCPKTGQVLANLELVPNYVLKNLISQWCEEN
Motif 10	29	42	GLPAMVQGVYSDDNALQLEATTQFRKLLS

Analysis revealed that Motif 1 and Motif 6 were present in 96 of the 98 ARM proteins, forming the core architecture of the ARM domain and typically spanning its N-terminal, central, and C-terminal regions (exceptions: AetARM5 lacked both motifs; TdARM15 lacked Motif 1). Motifs 2, 5, 7, and 8 were predominantly enriched in the C3 subfamily, occurring in 30, 35, 35, and 29 ARM members, respectively. Motifs 3 and 4 were primarily positioned flanking Motif 2: Motif 3 downstream and Motif 4 upstream. Additionally, Motif 9 was specifically distributed among 22 genes of the C2 subfamily, whereas Motif 10 was mainly present in both the C2 and C3 subfamilies (**Figure 2C**).

### 3.4 Gene duplication events and synteny analysis of *ARM* genes

**Figure 4:**
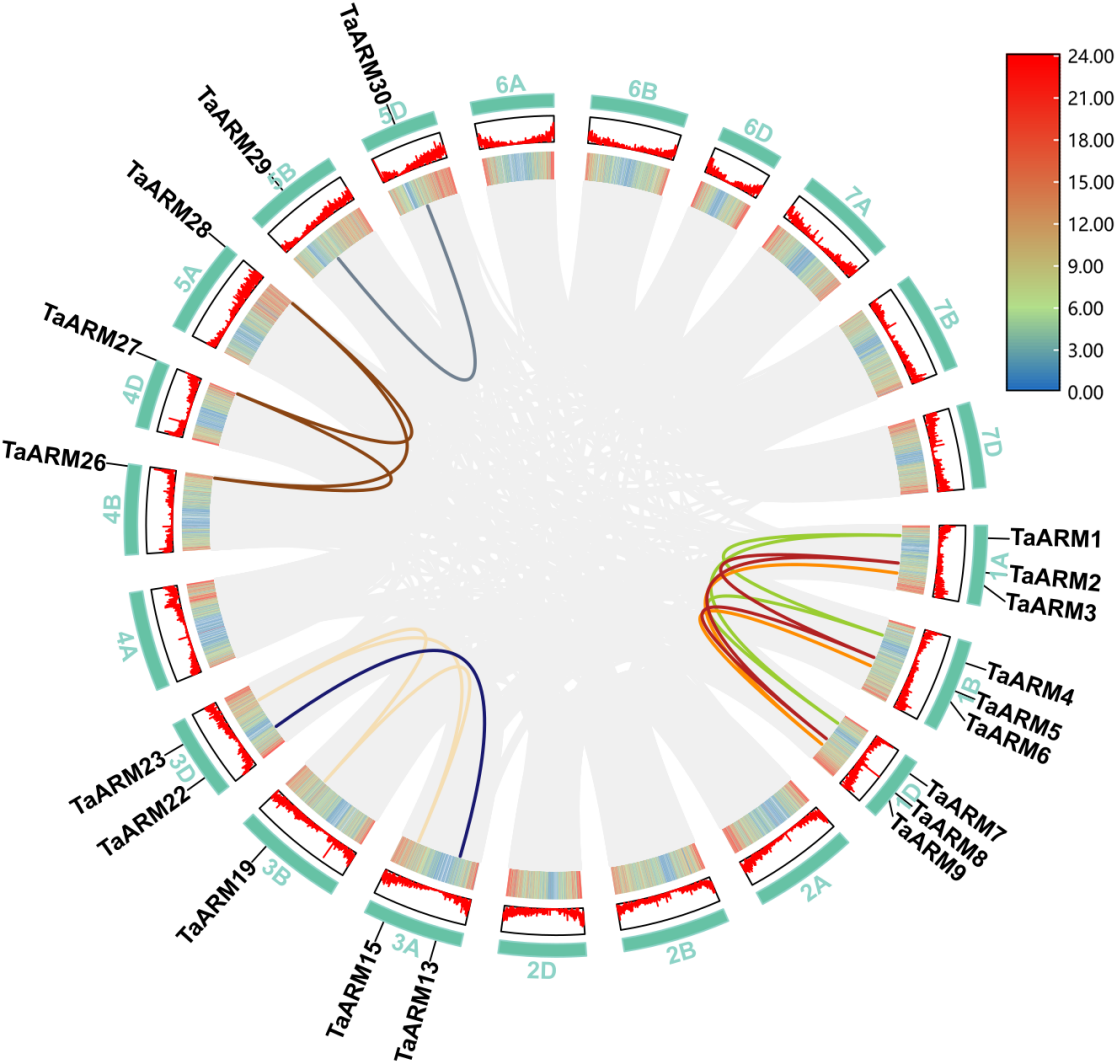
Gene duplication events in *TaARM* gene family. The chromosomes of wheat are depicted as teal circular arcs. The inner ring (Heatmap) and the middle ring (Line) both represent gene density along the chromosomes.

Polyploidy or whole−genome duplication, is recognized as a major driver of evolution and diversification in land plants (Panchy et al., 2016). Using the One Step MCScanX program, we identified 16 pairs of segmentally duplicated genes in the wheat *ARM* gene family, involving 19 *TaARM* genes (59.38% of the family; **Figure 4**). These pairs are as follows: *TaARM1*-*TaARM4*, *TaARM1*-*TaARM7*, *TaARM4*-*TaARM7*, *TaARM2*-*TaARM5*, *TaARM2*-*TaARM8*, *TaARM5*-*TaARM8*, *TaARM3*-*TaARM6*, *TaARM3*-*TaARM9*, *TaARM6*-*TaARM9*, *TaARM13*-*TaARM22*, *TaARM15*-*TaARM19*, *TaARM15*-*TaARM23*, *TaARM26*-*TaARM27*, *TaARM26*-*TaARM28*, *TaARM27*-*TaARM28*, and *TaARM29*-*TaARM30*. No tandem duplicates were detected in the wheat *ARM* gene family, suggesting that the expansion of *TaARM* genes likely arose from dispersed gene duplication events associated with wheat polyploidization. This mechanism appears to have played an important role in the expansion of the *TaARM* gene family.

The *Ka/Ks* is a widely used metric for inferring selective pressure (Vitti et al., 2013). For the 16 pairs of segmentally duplicated genes, *Ka*, *Ks*, and the *Ka/Ks* were computed using the Simple Ka/Ks Calculator (NG) tool. The calculated *Ka/Ks* values ranged from 0.000 to 0.263. Notably, three gene pairs (*TaARM1-TaARM4*, *TaARM2-TaARM8*, and *TaARM13-TaARM22*) exhibited a *Ka/Ks* ratio of zero. Since all *Ka/Ks* ratios are substantially less than 1, these results indicate that the *TaARM* gene family has predominantly undergone purifying selection. Purifying selection acts to remove deleterious mutations, thereby preserving the original structure and function of the encoded proteins (Hurst, 2002).

**Figure 5:**
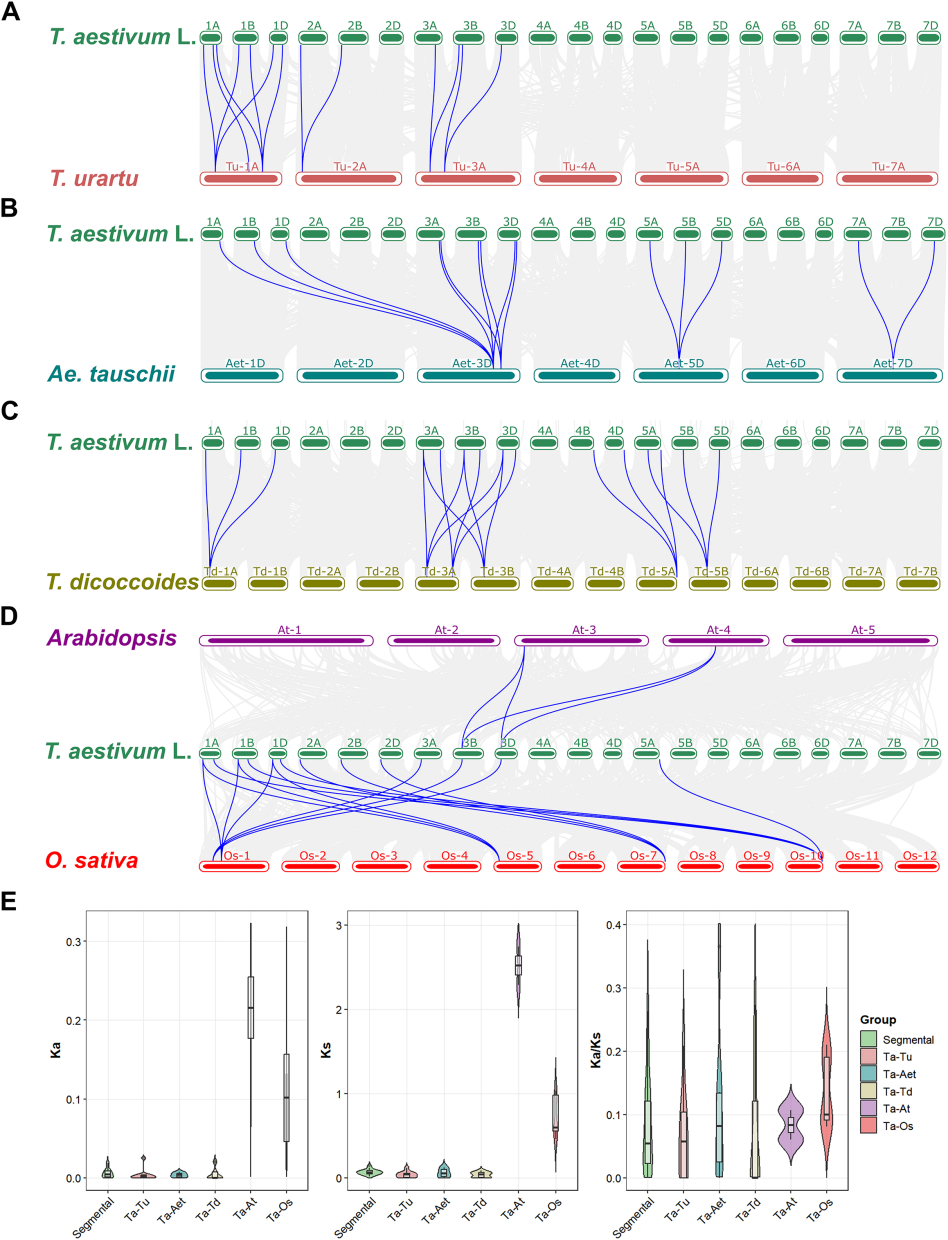
Synteny analysis and selection pressure analysis of *ARM* genes across multiple species. **(A–D)** display the interspecific synteny between wheat and its progenitors, as well as with *A. thaliana* and *O. sativa*. Different species are distinguished by distinct colors, and blue lines highlight the synteny-supported ARM gene pairs. Here, ‘Segmental’ denotes intraspecific segmental duplication events in wheat. **(E)** present the distributional features of *Ka, Ks*, and *Ka/Ks* values, respectively, within and among species, and compare the differences between groups. Each dataset is displayed using a box plot + violin plot combination, with different groups distinguished by different colors.

To investigate the evolutionary relationships of *ARM* genes between wheat and other species, synteny analysis was conducted using the One Step MCScanX program. This analysis identified 10 homologous gene pairs between common wheat (*Triticum aestivum* L.) and *T. urartu* with *Ka/Ks* ratios ranging from 0.000 to 0.209 (**Figures 5A, E**). Similarly, 6 homologous pairs were found between common wheat and *Aegilops tauschii* (*Ka/Ks*: 0.000–0.366; **Figures 5B, E**), and 13 pairs between common wheat and *T. dicoccoides* (*Ka/Ks*: 0.000–0.273; **Figures 5C, E**). Furthermore, 2 and 11 homologous gene pairs were identified between common wheat and *Arabidopsis thaliana* (*Ka/Ks*: 0.060–0.107) and rice (*Ka/Ks*: 0.081–0.210), respectively (**Figures 5D, E**). All calculated *Ka/Ks* ratios were below 1, indicating that purifying selection has been the predominant mode of selection acting on *ARM* genes during speciation. Detailed information on the syntenic gene pairs and their corresponding *Ka/Ks* ratios was provided in **Supplementary Table 3**.

### 3.5 The cis-acting regulatory elements analysis of *TaARM* genes

**Figure 6:**
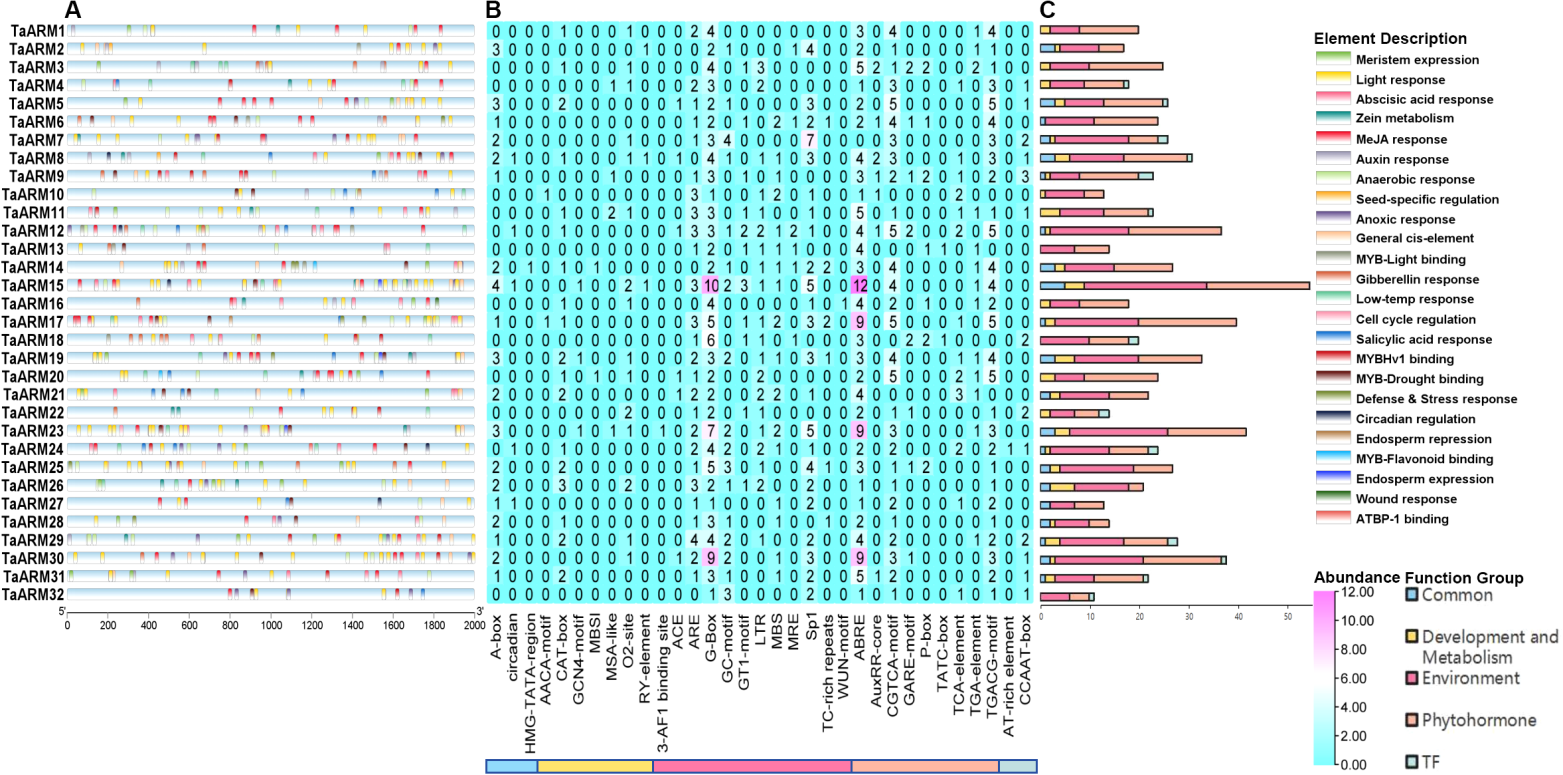
Cis-regulatory element composition of the *TaARM* gene family. **(A)** Distribution of cis-acting regulatory motifs in the promoter regions of *TaARM* genes. The color-coded legend “Element Description” corresponds to specific functional motifs (for original descriptions, refer to **Supplementary Table 5A**). **(B)** Heatmap illustrating the abundance of functional categories for the identified elements. The color scale represents the “Abundance” of each element. The digits indicate the count of cis-regulatory elements. **(C)** Stacked bar charts showing the classification of cis-elements into major functional groups. Different colors represent distinct “Function Groups” [Development and Metabolism, Environment, Phytohormone, and TF (Transcription Factor)].

To investigate the regulatory functions of *TaARM* genes under abiotic stress, we predicted cis-acting elements in the 2000-bp upstream regions of 32 *TaARM* genes based on the PlantCare database. The results showed that functionally diverse cis-acting elements are widely distributed upstream of these genes (**Figure 6A**; **Supplementary Table 5A**). According to functional annotations, we classified these elements into five major functional groups (**Supplementary Table 5B**). In the “Environment” group, we found a large number of elements involved in light response (e.g., G-box, Sp1), suggesting that *TaARM* genes may play a role in the regulation of light signaling and photosynthesis. Meanwhile, this group also contains a small number of elements related to environmental stress, such as LTR involved in low-temperature stress response and ARE essential for anaerobic induction, indicating that *TaARM* genes may have potential functions in defending against adverse environments (**Figure 6B**; **Supplementary Table 5B**). The “Phytohormone” group showed that *TaARM* genes have the ability to respond to various plant hormones. Among them, the ABRE element involved in abscisic acid response was the most abundant, followed by CGTCA-motif and TGACG-motif involved in MeJA (methyl jasmonate) response. These findings suggest that *TaARM* genes may participate in abiotic stress responses through complex hormone regulatory networks (**Figure 6B**; **Supplementary Table 5B**). We also assigned some transcription factor binding sites to the “Development and Metabolism” group; for example, MYB binding sites (MBSI) involved in flavonoid biosynthesis were found upstream of *TaARM14* and *TaARM20* (**Figure 6B**; **Supplementary Table 5B**). Notably, the upstream regions of all 32 *TaARM* genes were enriched with elements from the “Environment” and “Phytohormone” groups, suggesting that these genes may widely participate in abiotic stress responses (**Figure 6C**; **Supplementary Table 5C**). In addition, compared with widely distributed elements such as G-box and ABRE, some specifically or sparsely distributed elements (e.g., WUN-motif distributed only upstream of *TaARM16* and involved in wound response) may confer unique regulatory functions to certain *TaARM* genes.

### 3.6 Expression analysis of *TaARM* genes in different tissues of wheat

**Figure 7:**
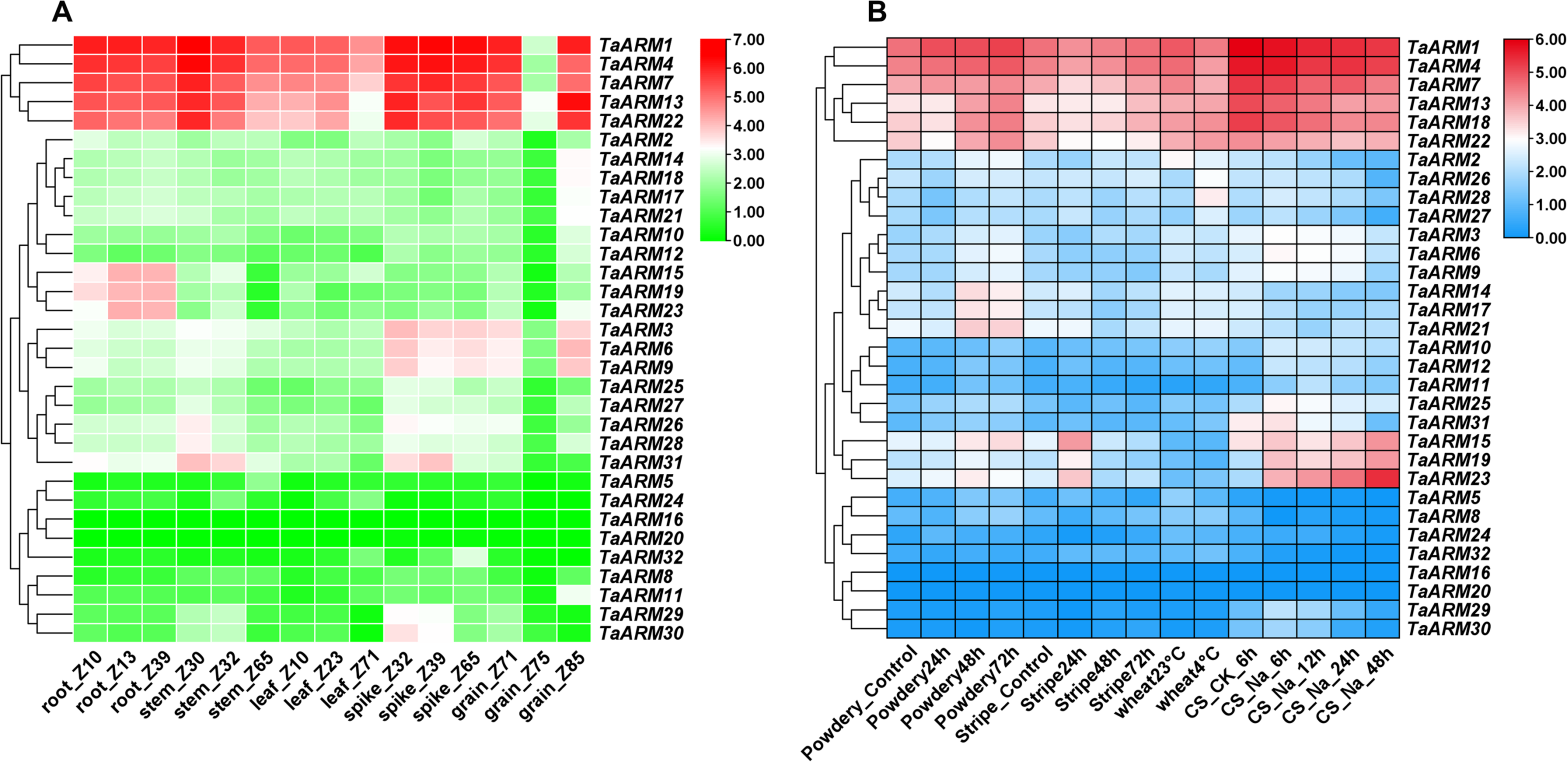
Expression pattern analysis of *TaARM* genes. **(A)** Expression levels analysis of *TaARM* genes in different tissues and developmental stages of wheat. Z, Zadoks decimal growth scale; Z + number denotes wheat developmental stage. **(B)** Expression levels analysis of *TaARM* genes under biotic and abiotic stresses. CS, Chinese Spring. Perform logarithmic transformation on the normalized TPM, and use ${\text{log}}_{2}(\text{TPM}+1)$ gene expression levels for visualization and differential analysis. log_2_(TPM + 1) > 3 indicates high gene expression levels, whereas 0 ≤ log_2_(TPM + 1) < 1 indicates low or no gene expression.

RNA-seq data for Chinese Spring were retrieved from the WheatOmics database (Ma et al., 2021b) to examine the expression profiles of *TaARM* genes across five tissues at multiple developmental stages. Expression analysis revealed that *TaARM1*, *TaARM4*, *TaARM7*, *TaARM13*, and *TaARM22* were consistently highly expressed (log_2_(TPM + 1) > 3) in all five tissues. In contrast, genes such as *TaARM16* and *TaARM20* showed low expression (0 ≤ log_2_(TPM + 1) < 1), suggesting limited functional roles during wheat growth and development. Several *TaARM* genes exhibited distinct tissue−specific expression patterns: *TaARM15*, *TaARM19*, and *TaARM23* were mainly expressed in the root; *TaARM26*, *TaARM28*, *TaARM29*, *TaARM30*, and *TaARM31* were mainly expressed in the spike; while *TaARM3*, *TaARM6*, and *TaARM9* showed expression in both spikes and grains (**Figure 7A**). These expression profiles imply that the corresponding *TaARM* genes may perform important, tissue−related functions during wheat development. The detailed data are provided in **Supplementary Table 7**.

### 3.7 Expression analysis of *TaARM* genes under biotic and abiotic stresses

In this study, RNA-seq data of wheat subjected to four distinct stress treatments were retrieved from the WheatOmics database (Ma et al., 2021b) to analyze the expression patterns of *TaARM* genes under the corresponding stress conditions. Genes with similar expression profiles were clustered together. We observed that *TaARM1*, *TaARM4*, *TaARM7*, *TaARM13*, *TaARM18*, and *TaARM22*, which grouped within the top high-expression subcluster, exhibited consistently elevated expression levels (log_2_(TPM + 1) > 3) across all stress conditions. Among these, *TaARM13*, *TaARM18*, and *TaARM22* displayed significant upregulation under powdery mildew stress. In contrast, most genes within the middle and low-expression subclusters maintained relatively low expression throughout the treatment period, indicating that they did not respond markedly to experimental stresses such as pathogen infection, salt, or cold treatment, and may represent constitutively low-expressed genes involved in basic cellular metabolic processes. Nevertheless, a subset of genes demonstrated stress-specific expression; for example, *TaARM15*, *TaARM19*, and *TaARM23* were highly expressed under salt stress, with notable upregulation during the late phase of treatment, suggesting their potential involvement in late-stage salt stress responses (**Figure 7B**). The detailed data are provided in **Supplementary Table 8**.

**Figure 8:**
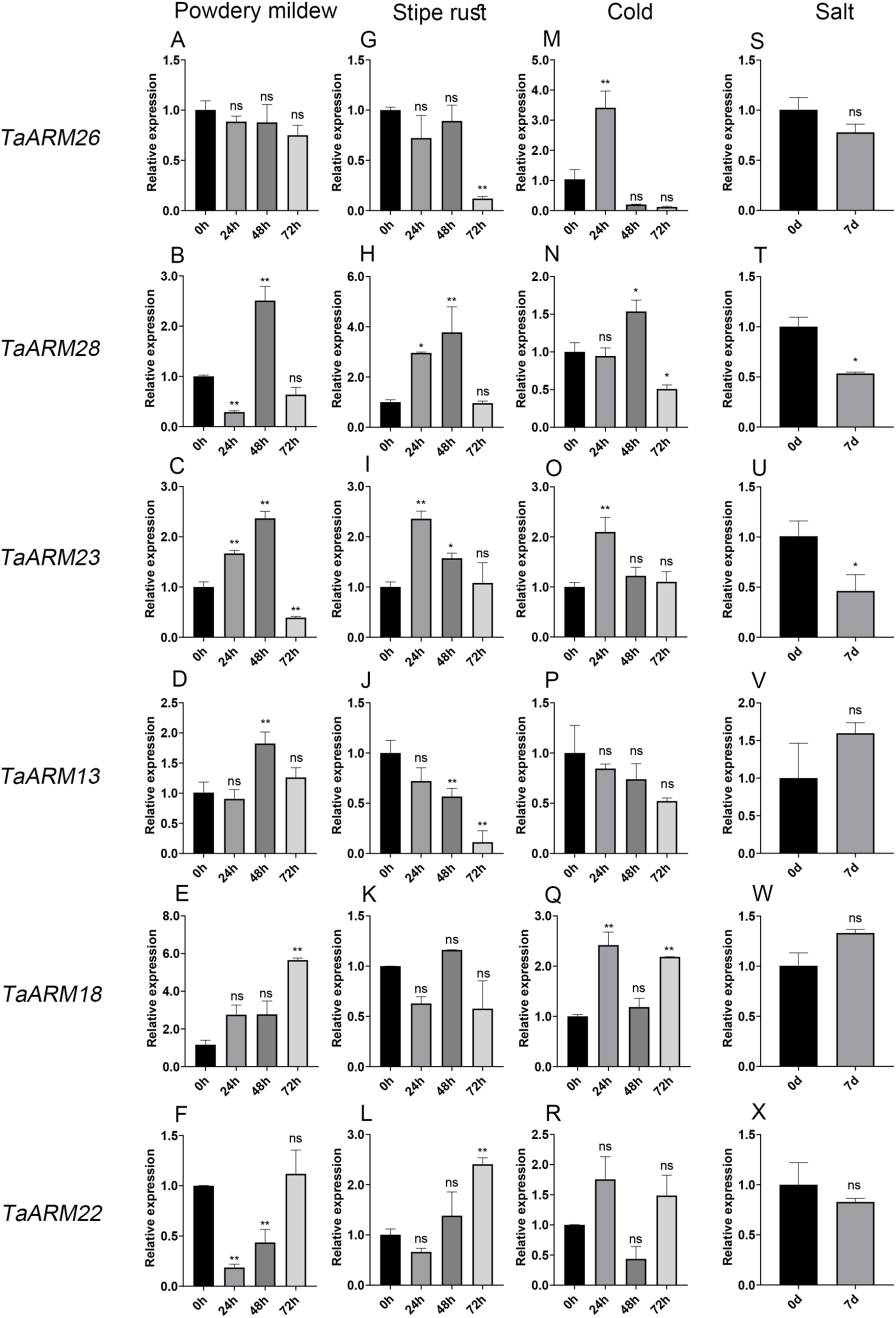
Expression levels of six *TaARM* genes in wheat under powdery mildew **(A–F)**, stripe rust **(G–L)**, cold **(M–R)**, and salt **(S–X)** stresses validated by qRT-PCR. The materials were treated with powdery mildew, stripe rust (CYR32), cold (4°C), salt (200 mmol/L NaCl). The materials under powdery mildew, stripe rust, and cold stress were sampled at 0h, 24h, 48h, and 72h, while materials under salt stress were sampled at 0d and 7d. Data represent mean ± standard error (SE) of three biological replicates. Statistically significant differences between the control and treatment groups are indicated using asterisks (ns, *p* ≥ 0.05; *p < 0.05, **p < 0.01, independent Student’s t-test).

To further investigate the functional roles of the ARM family under adverse conditions, the expression profiles of *TaARM26* and *TaARM28* (subfamily C1), *TaARM23* (subfamily C2), as well as *TaARM13*, *TaARM18*, and *TaARM22* (subfamily C3) were analyzed by qRT−PCR under powdery mildew, stripe rust, low-temperature, and salt stress treatments. As shown in **Figure 8**, these genes exhibited distinct expression patterns in response to the four stressors. *TaARM26* expression was significantly downregulated under stripe rust stress at 72 h but was upregulated under low−temperature stress at 24 h. Under powdery mildew stress, *TaARM28* displayed pronounced dynamic changes, showing significant upregulation at 48 h followed by downregulation at 72 h. During stripe rust stress, its expression was progressively upregulated at 24 h and 48 h, but decreased by 72 h. *TaARM23* expression was upregulated under powdery mildew, stripe rust, and low−temperature stresses at 24 h. *TaARM13* was upregulated under powdery mildew stress at 48 h, whereas it was downregulated under stripe rust stress at the same time point. *TaARM18* showed upregulation under powdery mildew stress at 72 h; under low temperature, its expression was elevated at 24 h, returned to baseline at 48 h, and increased again at 72 h. *TaARM22* was markedly downregulated at 24 h under powdery mildew stress before returning to basal levels by 72 h, while it was upregulated under stripe rust stress at 72 h. The differential responses of individual *TaARM* genes to various stress treatments suggest that they may perform distinct functional roles under different stress conditions.

## 4 Discussion

ARM repeats are widely distributed in plants, and their encoded proteins participate in key processes such as plant development, signal transduction, and responses to biotic/abiotic stresses (Samuel et al., 2006; Ni et al., 2010; Ngo et al., 2012; Mandal et al., 2017; Podia et al., 2018; Kong et al., 2019; Knop et al., 2021; Liu et al., 2024a). Although the *ARM* gene family has been identified in multiple species, genome-wide analysis of wheat (*T. aestivum* L.) remains insufficient. In this study, using bioinformatics methods, we identified 32 *ARM* genes in wheat​ and 8, 13, 22, 15, and 8 members in *T. urartu*, *Ae. tauschii*, *T. dicoccoides*, *A. thaliana*, and *O. sativa* ssp. *japonica*, respectively (**Supplementary Table 1**). Notably, substantial variation in *ARM* gene numbers exists across species under identical identification criteria. The number of *AtARM* and *OsARM* family members identified here differs from the results of Mudgil et al. (2004) and Sharma et al. (2014), which is primarily related to the setting of screening thresholds (**Supplementary Table 9**). Such discrepancies are normal, as the setting of screening thresholds should align with specific research objectives. Given that this study focuses on functional characterization, we adopted relatively stringent criteria to minimize the inclusion of false positives or truncated genes. Based on the phylogenetic tree of 98 *ARM* genes, we classified them into three subfamilies (C1-C3; **Figure 1**). Notably, the *ARM* genes of the C3 subfamily in wheat and its ancestral species exhibited a significant distribution bias on chromosomes, predominantly located on chromosomes 1–3 of the wheat A subgenome (1A, 2A, 3A), B subgenome (1B, 2B, 3B), and D subgenome (1D, 2D, 3D) (**Figure 3**). This distribution pattern is highly consistent with the polyploidization history of allohexaploid wheat (*T. aestivum*, AABBDD): its A and B genome donors *T. dicoccoides* (AABB) and D genome donor *Ae. tauschii* (DD) hybridized and naturally doubled to form allohexaploid wheat (Zhao et al., 2023). Intraspecific collinearity analysis further revealed that *ARM* genes exhibit numerous collinear blocks among the A/B/D subgenomes, indicating that chromosomal segment duplication accompanying polyploidization events is the primary mechanism driving the expansion of this gene family (**Figure 4**). The research findings support the polyploidization-driven gene family expansion model, providing new evidence for elucidating the evolution and functional differentiation of wheat *ARM* genes.

Furthermore, multi-species synteny analysis revealed high evolutionary conservation among certain *ARM* genes. For instance, *TaARM1* is orthologous to *TuARM1* and *TdARM1*, *TaARM24* to *AetARM9*, *TaARM22* to *AtARM6* and *OsARM1*. These orthologous genes cluster within the same phylogenetic clade, share identical gene structures, and display conserved motif composition and organization, implying functional similarity across diverse plant species (**Figures 2A, B**). Calculation of the *Ka/Ks* ratio for each syntenic gene pair indicated that *ARM* gene family members are predominantly under purifying selection, which removes deleterious mutations and preserves protein structure and function (**Supplementary Table 3**) (Hurst, 2002), thereby reinforcing the evolutionary conservation of this gene family. Similar patterns of conservation have been reported for the *ARM* gene family in other crops, including potato (Liu et al., 2024b) and maize (Yu et al., 2023).

In this study, we retrieved RNA-Seq data from the WheatOmics database to examine wheat responses under diverse biotic (powdery mildew and stripe rust) and abiotic (low temperature and salt) stresses. Clustering analysis of *TaARM* genes based on expression profiles revealed that *TaARM1*, *TaARM4*, *TaARM7*, *TaARM13*, *TaARM18*, and *TaARM22* maintained relatively high expression levels across all stress conditions (**Figure 7B**). Notably, *TaARM1*, *TaARM4*, *TaARM7*, *TaARM13*, and *TaARM22* were also highly expressed in five wheat tissues (root, stem, leaf, spike, and seed), suggesting potential dual roles in both stress adaptation and growth/development (**Figure 7A**). Using qRT-PCR, we validated the expression of six selected genes (*TaARM26*, *TaARM28*, *TaARM23*, *TaARM13*, *TaARM18*, and *TaARM22*) under each of the four stress treatments (**Figure 8**). The results demonstrated that these genes displayed distinct response profiles to different stresses, and individual *TaARM* genes often responded differentially depending on the stress type, indicating that they may perform context-specific roles under varying environmental conditions. Cis-acting element analysis revealed that the promoter regions (2 kb upstream) of these *TaARM* genes are enriched with numerous environment- and hormone-responsive cis-regulatory elements, including ABRE elements involved in abscisic acid (ABA) signal transduction, G-box associated with light response, LTR related to low-temperature stress response, and CGTCA-motif and TGACG-motif regulated by jasmonic acid signaling (**Supplementary Table 5**). The significant enrichment of these elements suggests that *TaARM* genes may participate in wheat’s response to abiotic stresses such as drought and high salinity by integrating ABA signaling, light regulation, and hormonal responses (**Figures 6B, C**). Furthermore, the coexistence of multiple stress-responsive elements indicates the potential formation of a complex regulatory network that synergistically modulates gene expression. This finding provides critical insights into the molecular regulatory mechanisms of *TaARM* genes in abiotic stress responses.

Based on existing research, *ARM* family genes are likely to play crucial roles in plant stress resistance. For instance, *Arabidopsis thaliana ARM* family members *PUB20* and *PUB21*, when present as loss-of-function mutants, exhibit enhanced resistance to bacterial pathogens, with significantly reduced pathogen counts following inoculation (Yi et al., 2024). In *Tomato leaf curl New Delhi virus* (ToLCNDV)-resistant varieties, the *ARM* family gene *SlARM18* shows marked upregulation, while virus-induced gene silencing (VIGS)-mediated downregulation of this gene results in exacerbated disease symptoms, suggesting its potential involvement in antiviral responses (Mandal et al., 2017). In cotton, knockout of *GhARM* enhances plant resistance to Verticillium wilt and alleviates associated disease symptoms (Zhang et al., 2025b). These findings across diverse species indicate that *ARM* family genes may exhibit functional conservation in plant stress resistance mechanisms. Therefore, we hypothesize that wheat *TaARM* genes may also play comparable roles in stress responses. The functional characterization of a gene family is governed not only by its genetic sequence, but also by protein-protein interactions, involvement in metabolic pathways, and additional regulatory layers. To systematically characterize the biological functions of the *TaARM* gene family in wheat, we will integrate multi-omics analyses with molecular validation and transgenic functional verification. This comprehensive approach aims to establish a molecular foundation for developing stress-resistant wheat cultivars.

## 5 Conclusions

In this study, we identified and characterized *ARM* genes across wheat and related species. In hexaploid wheat, the family expanded via polyploidy-driven segmental duplications and evolved under purifying selection. The abundance of cis-acting elements upstream of *TaARM* genes endows them with the potential to regulate abiotic stress responses. *TaARM* genes exhibit tissue-specific and stress-responsive expression, with qRT-PCR confirming their stress-specificity, indicating specialized roles in a coordinated network. The qRT-qPCR results demonstrated that *TaARM13* and *TaARM22* respond specifically to biotic stresses (powdery mildew and stripe rust). *TaARM13* was up-regulated in response to powdery mildew stress but down-regulated under stripe rust stress, whereas *TaARM22* exhibited up-regulation under all tested biotic stresses. *TaARM18* showed specific responses to powdery mildew infection and low-temperature stress, with significantly up-regulated expression under both stresses. In contrast, *TaARM26* was specifically responsive to stripe rust infection (with down-regulated expression) and low-temperature stress (with up-regulated expression). This study provides a foundation for leveraging *TaARM* genes in breeding stress-resilient wheat.

## Data Availability

The original contributions presented in the study are included in the article/**Supplementary Material**. Further inquiries can be directed to the corresponding authors.
